# Supplementary material for: Treatment burden experienced by patients with lung cancer
Source: PLoS One. 2021 Jan 22;16(1):e0245492. doi: 10.1371/journal.pone.0245492 (PMC7822249; doi:10.1371/journal.pone.0245492)
Supplement: S1 File — (DOCX) [file pone.0245492.s001.docx]

Interview Question Guide

| **Topic** | **Question(s)** |
| --- | --- |
| Introduction | Introduce definition of treatment burden for patient  “Treatment burden is the workload that you have to take on in order to complete treatment. This can include things like travel, waiting times, changing lifestyle, organizing care, etc.” |
| General Treatment Burden | “Based on the definition I gave you, can you list some things you have found burdensome since beginning treatment? This can include tasks that your doctor has assigned to you or other things you have had to take on in order to complete those tasks.” |
| Healthcare Provider Assigned Tasks | “What are some things that your doctor or other health professionals have prescribed or told you to do for your treatment? – This can include suggestions or advice from any health care professional”  “Have you been prescribed any medications, diets or exercise regimes for your treatment? Have you had to organise treatment, follow up appointments or scans yourself? Have you had to learn any new information about your disease or treatment? Have you been asked to not do anything, like smoke, eat particular foods, go to particular places or travel, etc.?”  “How much of a burden/hassle has this been for you? [Not at all, somewhat, neutral, burdensome, very burdensome]” |
| Goals | “What are the goals of your treatment? OR  What do you aim to achieve from treatment? OR  Why did you decide to do this treatment?”  Can you talk me through the decision making process when you agreed to do the treatment” |
| Workload to Complete Healthcare Provider Assigned Tasks | “What are some other jobs you have had to take on to be able to complete the prescribed tasks? Have you had to organise care for yourself or others? organise transport? Pay for treatments up front? Complete/manage any paperwork? Has it been difficult to learn about and navigate the healthcare system?”  “How much of a burden/hassle has this been for you? [Not at all, somewhat, neutral, burdensome, very burdensome]” |
| Time | “Consider a typical day when you come in for the therapy. How much time do you spend organising to complete the therapy, travelling, parking, in the waiting room and receiving therapy? In a week, how much time do you think you spend for your treatment (including getting and organising medications and treatments, travel, time lost from side effects from treatment, etc.)”  “Do you feel like this time spent for treatment is taking away from your life or is it just part of what you need to do? In other words, the you described; did it feel like that time was lost to treatment?” |
| Treatment Consequences | “Thinking of your treatment and side effects and follow up, did you lose anything, or have to give something up (can include time, relationships, work/social time, smoking, etc). Is there anything you have had to take on additionally to complete your treatment? (including additional medications to manage side effects, extra work to make up for time lost, etc)  “Have you had to pay for anything other than the treatment itself, to complete treatment? (travel/accommodation costs, additional therapy to manage symptoms or emotional wellbeing, formal care for someone, etc.)” |
| Impact on Daily Life | “Have you experienced a financial burden from your treatment? Can be from additional cost and lost income”  “Has your treatment impacted your emotional wellbeing?”  “How much of an impact has your treatment had on your appetite and desire to complete usual activities or socialise?”  “Has your treatment affected your sleep?”  “How much of an impact has treatment had on your home situation (cooking, cleaning, chores)/work/social life (going out, spending time with family/friends)?” |
| Impact on Relationships | “How would you describe your relationship with your healthcare providers? (Do you feel comfortable discussing intimate details of your health and wellbeing (emotional and mental health)? Why/why not?)”  “How about with your family and friends? (Have your relationships changed for better/worse?)”  “Have you had to do anything to maintain or improve your relationships? (Some people have described having to Lie, avoid people, force yourself to socialize, etc.)”  “Do you feel any expectations from others?”  “To what extent do you feel that your treatment has affected (or even burdened) others? Have other family members or friends or colleagues had to pick up tasks?” |
| External Exacerbating Factors | “Have there been other social or personal circumstances that have worsened your experience with completing your treatment? (Personal/religious beliefs, societal expectations, etc.)”  “People have described that there is a particular opinion or stigma towards lung cancer and people who have it. Would you agree with that idea? Do you think you have felt that perception directed to you? |
| Needs | “a lot of people find that during treatment, they have needs that are not met with the current system. Can you think of a time where you felt you had a need that wasn’t met? This could be a time where you thought ‘oh, *this* would be helpful right now.” |
| Closing | “Overall, would you describe your treatment as burdensome?”  “Is there anything you believe is important that I haven’t asked about?” |
